# Lipid-Laden M1-like Macrophages of Familial Hypercholesterolemia Patients Are Characterized by Increased Interleukin-1β Secretion and Reduced *TMEM176A* and *TMEM176B* Gene Expression

**DOI:** 10.3390/cimb48080769

**Published:** 2026-07-29

**Authors:** Artem Izyumchenko, Tatiana Usenko, Kseniia Dracheva, Kristina Legostaeva, Soreiia Urazgildeeva, Kseniia Tanayants, Maria Grunina, Ekaterina Larionova, Pavel Suchko, Oleg Glotov, Alexandr Kulikov, Sofya Pchelina, Valentina Miroshnikova

**Affiliations:** 1 Department of Molecular Genetic and Nanobiological Technologies, Pavlov First Saint-Petersburg State Medical University, 197022 Saint-Petersburg, Russia; artemiz98@yandex.ru (A.I.);; 2 Petersburg Nuclear Physics Institute Named by B.P. Konstantinov of National Research Centre «Kurchatov Institute», 188300 Gatchina, Russia; 3 Department of Propaedeutics of Internal Diseases, Pavlov First Saint Petersburg State Medical University, 197022 Saint-Petersburg, Russia; 4 Saint-Petersburg Medical and Social Institute, 195271 Saint-Petersburg, Russia; 5 Federal State Budgetary Research Institution “Institute of Experimental Medicine”, 197022 Saint-Petersburg, Russia; 6 Centre for Genomic Technologies Cerbalab, 199106 Saint-Petersburg, Russia; 7 Pediatric Research and Clinical Center for Infectious Diseases Under the Federal Medical Biological Agency, 197022 Saint-Petersburg, Russia

**Keywords:** familial hypercholesterolemia, M1-like macrophages, RNAseq, interleukin-1β, chemokine signaling, TMEM176, inflammasome

## Abstract

Familial hypercholesterolemia (FH) is a common hereditary dyslipidemia characterized by high levels of low-density lipoprotein (LDL) cholesterol and a risk of premature atherosclerosis. Previous studies showed that cardiovascular risks persist in FH even after hypolipidemic therapy is started and are linked to a proinflammatory status of circulating monocytes. In the current study we aimed to identify FH-specific gene expression patterns of monocyte-derived M1-like macrophages in response to lipid accumulation. RNAseq was performed for four patient and four control paired samples of M1-like macrophages before and after incubation with oxidized LDL (oxLDL). A validation step was performed in 10 patients and 10 controls using real-time PCR and ELISA. RNAseq data analysis revealed 22 DEGs between FH patients and the control group before and 47 DEGs after incubation with oxLDL. Pathway enrichment analysis suggested dysregulation of inflammatory pathways especially IL-1 and chemokine signaling in response to lipid accumulation in FH M1-like macrophages. Validation experiments demonstrated increased interleukin-1β secretion by lipid-laden M1-like macrophages in FH patients and reduced *TMEM176A* and *TMEM176B* gene expression compared to controls. Our results suggest FH M1-like macrophages may be predisposed to an accelerated immune response via interleukin-1β due to reduced TMEM176A/B activity.

## 1. Introduction

Familial hypercholesterolemia (FH) is the most common hereditary dyslipidemia characterized by high levels of total cholesterol (TC) and low-density lipoprotein cholesterol (LDL-C) from birth that results in subsequent development of premature atherosclerosis and cardiovascular disease (CVD). Undiagnosed FH is known to lead to prolonged exposure to elevated LDL levels, which promotes the oxidation of LDL to oxLDL [1]. These oxLDL particles are taken up by intimal macrophages, contributing to the initiation of atherosclerotic plaque formation. This process is characterized by non-resolving inflammation and the recruitment of additional immune cells into the vessel wall. Despite the successes achieved by lipid-lowering therapy, cardiovascular risks persist in mutation carriers [2]. Previous studies suggested that “trained immunity” might be a mechanism that contributes to increased cardiovascular risk retention in patients with FH [2,3,4].

Signs of inflammation in FH appear early in childhood: children with FH exhibit increased serum levels of TNFα and decreased levels of IL-10, which contribute to a state of non-resolving inflammation [5]. Congenital disturbance of lipid homeostasis in FH is characterized by myelomonocytic skewing and a shift in monocyte subpopulations, with an increased proportion of higher circulating proinflammatory and non-classical monocytes [6,7,8]. Stiekema et al. assumed that these FH special features of the monocyte population could be linked to early changes in lipid homeostasis of bone marrow (BM) hematopoietic stem and progenitor cells (HSPCs) [8]. BM HSPCs and subsequently monocytes from patients with FH show an increased number of lipid droplets indicating the beginning of intracellular lipid accumulation [8,9,10]. Transcriptomic profiling showed that a large number of genes associated with inflammation and cell migration, belonging to the vascular endothelial growth factor and chemokine families, are activated in BM HSPCs in untreated FH patients. Moreover, the expression of genes involved in monocyte and macrophage-mediated inflammation and migration in BM HSPCs remains elevated after lipid-lowering treatment [8]. This may explain the observed increase in percentage of “M1 monocytes” which are predisposed to differentiate into M1 macrophages in FH [2,3,11,12].

Macrophages are the predominant immune cells in atherosclerotic lesions and single-cell analyses have revealed demonstrated macrophage heterogeneity within atherosclerotic plaques [13,14]. M1 macrophages, M2 macrophages, and intermediate M1/M2 macrophages form three main macrophage clusters [14]. Additional plaque-specific macrophage phenotypes were recently identified, including Mhem, Mox, and M4; therefore, plaque macrophages require a more specific classification and the M1/M2 definition is likely insufficient [15]. Notably, one subset of plaque macrophages resembles “classically activated” M1 macrophages derived from newly recruited monocytes and is characterized by high expression of proinflammatory genes, including *IL1B* and *TNFA*, as well as various chemokines [13,16]. These inflammatory M1 macrophages drive lesion progression by secreting classical proinflammatory cytokines, which amplify local inflammation and recruit additional immune cells.

**Figure 1:**
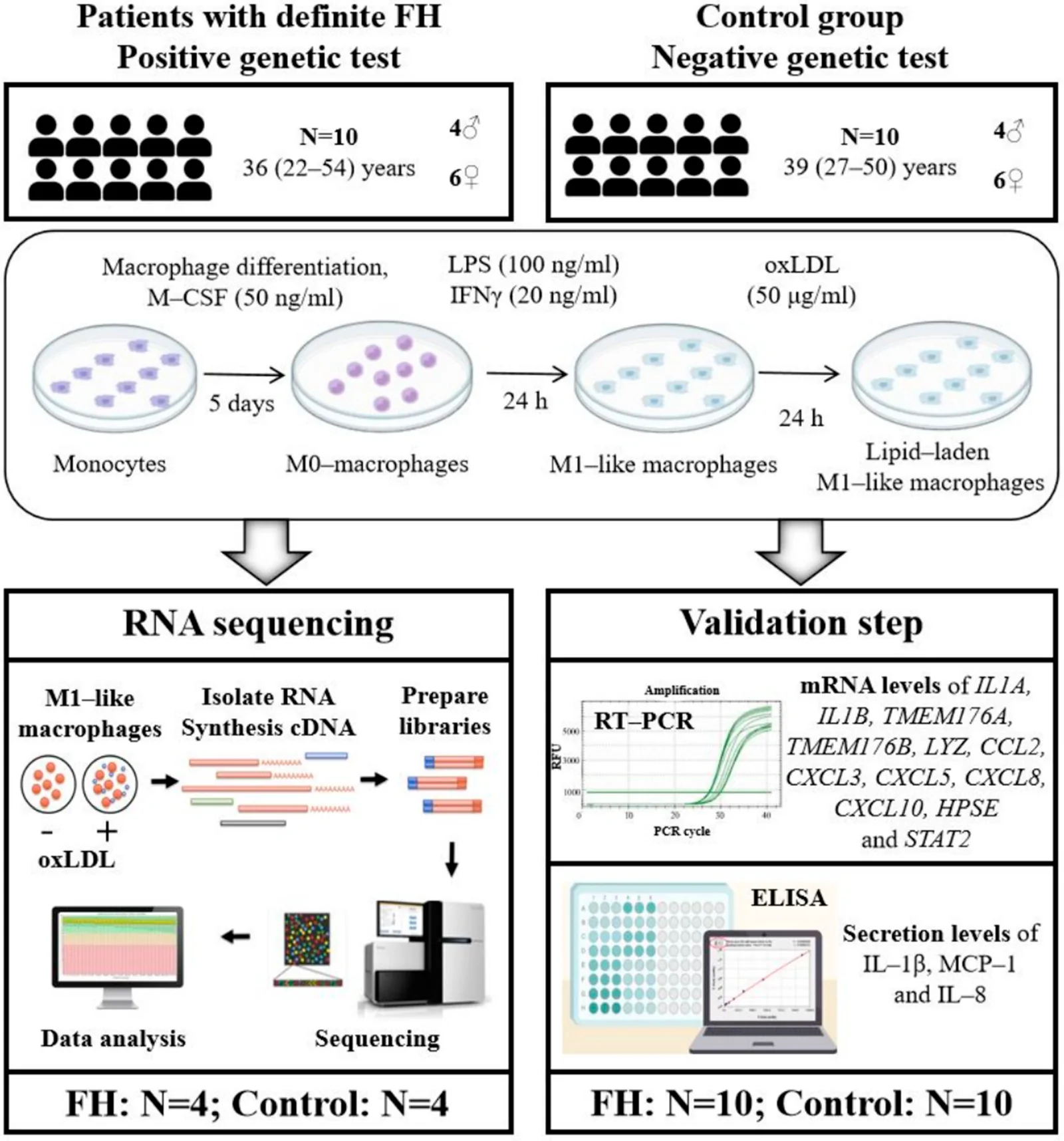
Study design.

FH monocytes are prone to polarization toward the M1 phenotype; thus, it can be hypothesized that preactivated FH M1 macrophages may demonstrate increased inflammatory responses due to their “trained immunity”. Previously we described the spectrum of mutations in FH patients in Saint-Petersburg, Russia [17,18]. Here we performed RNA-seq of monocyte-derived M1-like macrophages in patients with genetically confirmed FH and in a control group. Incubation with oxidized low-density lipoproteins (oxLDLs) was used to imitate lipid overloading and macrophage foam cell formation. The study design is shown in Figure 1.

## 2. Materials and Methods

### 2.1. Participants

Ten FH patients from Pavlov First Saint-Petersburg State Medical University were recruited for this study. The inclusion criteria included a definite FH diagnosis according to the Dutch Lipid Clinic Network (DLCN) criteria (a total score of greater than 8 points) and a positive genetic test: 8 patients heterozygous for different pathogenic variants in the *LDLR* gene and 2 patients heterozygous for the p.Arg3527Gln variant in the *APOB* gene were included (Table 1). Patients were treated according to their physician with rosuvastatin and ezetimibe (Table 1). Ten age-matched controls were recruited. The criteria for inclusion in the control group included absence of dyslipidemia and CVD and a negative genetic test. The study protocol is in accordance with the Declaration of Helsinki and was approved by the local ethics committee of Pavlov First Saint-Petersburg State Medical University, Saint-Petersburg, Russian Federation (protocol 298 on 31 March 2025). Written informed consent was given by each participant.

### 2.2. Human Monocyte-Derived M1-like Macrophages

Peripheral blood mononuclear cells (PBMCs) were obtained from 50 mL of freshly collected whole blood by gradient centrifugation at 1600 rpm in Ficoll solution (PanEco, Moscow, Russia). The obtained mononuclear cells were transferred into 6-well plates in culture medium (RPMI 1640, 2 mM L-glutamine, 10% Fetal Bovine Serum (FBS) and 1% gentamicin (all PanEco, Moscow, Russia)) at a rate of at least 5 × 10^6^ cells per well (1 mL of suspension) and incubated in a CO_2_ incubator at +37 °C for 2 h. Then, attached monocytes were cultured further in the presence of macrophage colony-stimulating factor 50 ng/mL M-CSF (Biolegend, San Diego, CA, USA) for 5 days (M0-macrophages) with daily change in medium at an average density of 2 × 10^5^ cells per well. Cell counting was performed using an automatic cell counter TC20 (BioRad, Hercules, CA, USA). For M1 polarization induction, M0-macrophages were next exposed to a fresh medium supplemented with 5% FBS and containing lipopolysaccharide 100 ng/mL LPS (Medgamal, Moscow, Russia) and 20 ng/mL IFNγ (FineTest, Wuhan, Hubei, China) for an additional 24 h [19]. Macrophage phenotype was confirmed by flow cytometry and real-time PCR according to Jaguin et al. [19] (Figures S1 and S2).

### 2.3. oxLDL Preparation and Macrophage Lipid Loading

Human LDLs (density = 1.023–1.055 g/mL) were isolated from the blood plasma of healthy donors by NaBr density gradient centrifugation as described previously [20,21]. Oxidation of LDL was performed by dialysis against EDTA-free isotonic saline supplemented with 4 MM CuSO_4_ at 37 °C for 18 h [21]. Lipid oxidation products were detected by the malondialdehyde assay using thiobarbituric acid as described [22]. Lipoprotein protein concentration was determined by the Lowry method. M1-like macrophages were incubated with oxLDLs at a concentration of 50 μg lipoprotein protein per ml of culture medium (RPMI 1640, 2 mM L-glutamine, 10% lipid-depleted serum (Biowest, Nuaillé, France) and 1% gentamicin) for an additional 24 h. Lipid accumulation in macrophages was confirmed by Oil Red O staining.

### 2.4. RNAseq

Paired samples of M1-like macrophages before and after incubation with oxLDLs for four patients and four controls (marked in Table 1; total 16 samples) were subjected to RNAseq. Total RNA was isolated using the RNeasy mini kit according to the manufacturer’s instructions (Qiagen, Valencia, CA, USA). RNA quality was assessed with NanoDrop 1000 Spectrophotometer (Thermo Fisher Scientific, Gothenburg, Sweden) and agarose gel electrophoresis; RNA quantity—Equalbit RNA HS Assay Kit (Vazyme, Nanjing, China). A total of 250 ng of total RNA was used for double-stranded cDNA synthesis with Oligo-dT priming using the Mint kit (Eurogen, Moscow, Russia). Double-stranded cDNA was then subjected to enzymatic fragmentation, and further construction of DNA libraries was proceeded using the VAHTS Universal V8 RNA-seq Library Prep Kit for MGI (Vazyme, Nanjing, China) according to the manufacturer’s instructions. Library quality was assessed with Tape Station 4200 (Agilent, Waldbronn, Germany); quantity—QuDye^®^ dsDNA HS (Lumiprobe, Moscow, Russia). Sequencing was performed on a DNBSEQ-400 (MGI, Shenzhen, China) instrument using an FCL high-throughput sequencing set flow cell (PE100, 360 Gb) in paired-end read mode.

### 2.5. Quality Control and Read Preprocessing

Quality control for each sample was performed using FastQC (v0.11.9). Adapter trimming and read preprocessing were carried out using Trimmomatic (v0.38) for paired-end sequencing data. During preprocessing, low-quality reads, reads containing ambiguous bases, and adapter-contaminated reads were removed. Paired-end trimmed reads were generated for each sample and used for all downstream analyses. FastQC reports were used to assess sequencing quality metrics, including per-base sequence quality, GC content and potential adapter contamination. Only paired-end trimmed reads were retained for downstream analyses.

### 2.6. Read Alignment to the Reference Genome

High-quality reads were aligned to the human reference genome (GRCh38 primary assembly, version GRCh38.p14, https://www.gencodegenes.org/human/, accessed on 2 April 2026) using HISAT2 (v2.2.1). Gene-level read quantification was performed using HTSeq-count (v2.0.4) with the GENCODE v50 gene annotation (GRCh38). Read counts were summarized at the gene level using the gene_id attribute. The resulting count matrices were used for differential gene expression analysis.

### 2.7. Differential Gene Expression Analysis

Differential gene expression analysis was performed using the DESeq2 package (v1.32.1) in R (4.3.2). A DESeqDataSet object was constructed using a multifactor experimental design including biological group and treatment variables, as well as their interaction term (design = ~Group + oxLDL + Group:oxLDL). Prior to analysis, genes with low counts (sum of reads ≤ 10 across all samples) were removed to reduce noise and improve statistical robustness. Differential expression analysis was performed using Wald tests implemented in DESeq2. Contrasts were defined according to the experimental design and included comparisons between FH and control groups under different treatment (oxLDL) conditions, oxLDL effects (Yes vs. No) within each group and interaction effects between group and oxLDL. Genes with an absolute log2foldchange ≥ log2(1.5) and *p*-values < 0.05 were considered as significantly differentially expressed genes (DEGs). Gene annotation was performed using AnnotationDbi (v1.64.1) and org.Hs.eg.db (v3.18.0).

### 2.8. Functional Enrichment Analysis

Gene Ontology (GO) enrichment analysis of differentially expressed genes was performed using the Gene Ontology resource (http://geneontology.org, accessed on 9 April 2026). Functional enrichment and network analysis were conducted using ClueGO (v2.5.7) plugin for Cytoscape (v3.6.1). GO terms with adjusted *p*-value < 0.05 were considered significantly enriched. Term grouping in ClueGO was based on shared gene overlap (>50%). Term clusters were defined based on common gene associations. A functional interaction network of selected biological processes and differentially expressed genes was constructed using CluePedia.

### 2.9. Real-Time PCR

Total RNA was isolated using the RNeasy mini kit according to the manufacturer’s instructions (Qiagen, Valencia, CA, USA). RNA quality was assessed with NanoDrop 1000 Spectrophotometer (Thermo Fisher Scientific, Gothenburg, Sweden) and agarose gel electrophoresis; RNA quantity—Equalbit RNA HS Assay Kit (Vazyme, Nanjing, China). First-strand cDNA was synthesized using the Magnus reverse transcriptase (Eurogen, Moscow, Russia). mRNA levels of *IL1A*, *IL1B*, *TMEM176A*, *TMEM176B*, *LYZ*, *CCL2*, *CXCL3*, *CXCL5*, *CXCL8*, *CXCL10*, *HPSE*, and *STAT2* genes were determined by real-time PCR with TaqMan PCR Master Mix (AlcorBio, Saint-Petersburg, Russia) or SYBR Green Master Mix (Eurogen, Moscow, Russia) on the CFX96 device (BioRad, Hercules, CA, USA). Threshold cycle (Ct) values were obtained and relative gene expression was normalized to two reference genes (*ACTB* and *RPLP0*). The primer and probe sequences used in this work are presented in Table S1.

### 2.10. ELISA

Secretion levels of IL-1β encoded by *IL1B* gene, monocyte chemoattractant protein-1 (MCP-1) encoded by *CCL2* gene and IL-8 encoded by *CXCL8* gene were determined in culture supernatants using commercial ELISA kits (Vector Best, Novosibirsk, Russia).

### 2.11. Statistical Analysis

Statistical analyses were performed using R software (version 4.3.2) with built-in and pre-installed packages. Data normality was assessed using the Shapiro–Wilk test. Pairwise comparisons between independent groups (FH patients vs. controls) were conducted using the two-tailed Wilcoxon rank-sum test (Mann–Whitney U test) (or *t*-test in the case of normal distribution), while comparisons between related groups (before and after incubation with oxLDLs) were performed using the two-tailed Wilcoxon signed-rank test. A *p*-value < 0.05 was considered statistically significant. Clinical and experimental values are presented as median (minimum–maximum) (or mean ± SD in the case of normal distribution).

## 3. Results

The demographic and biochemical characteristics of the participants, including serum IL-1β, MCP-1 and IL-8 concentrations, are summarized in Table 2. The groups were similar regarding age and sex. Individual characteristics (mutations, lipid levels and therapy) are presented in Table 1.

### 3.1. Transciptomic Profiling of M1-like Macrophages

**Figure 2:**
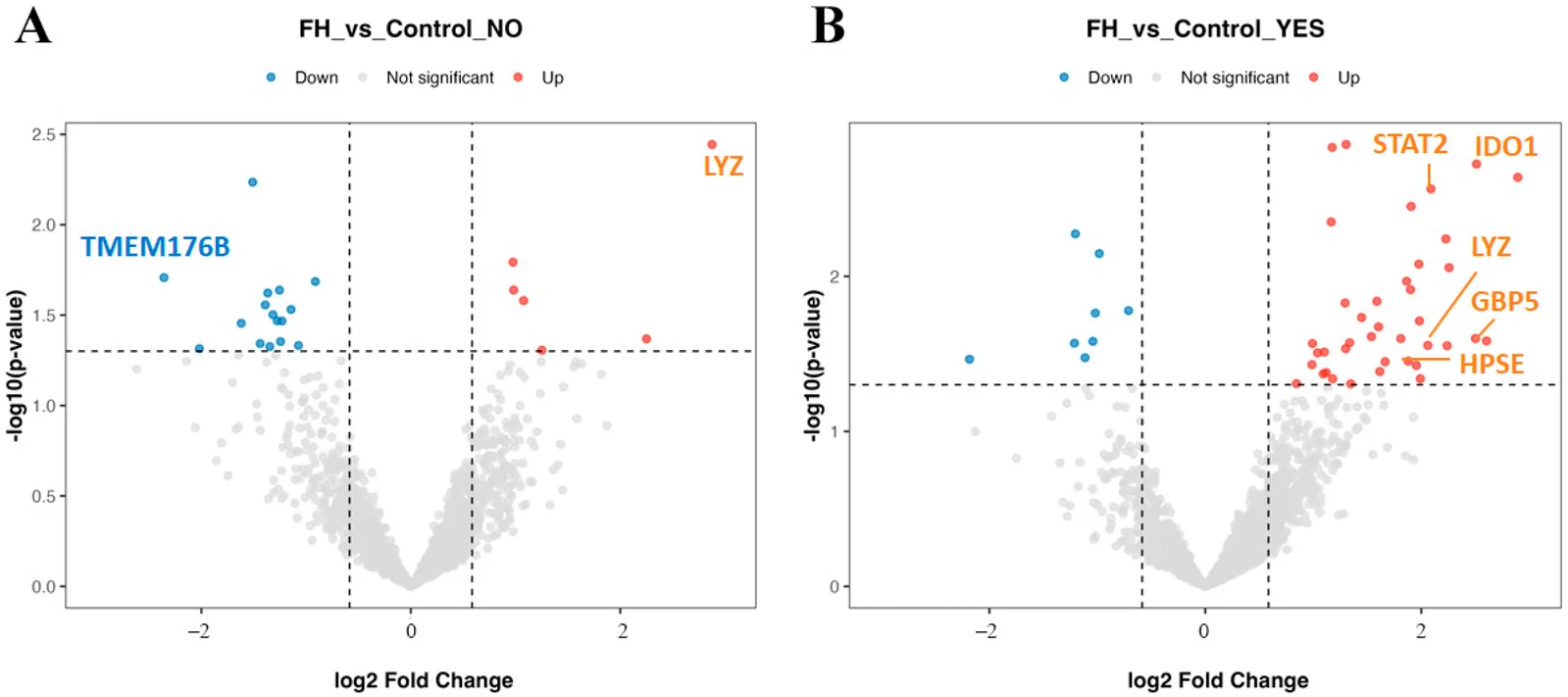
Transcriptomic profiling of M1-like macrophages of FH patients: volcano plot of mRNA expression in FH versus controls constructed using log2 fold-change (|FC| > 1.5) values and *p*-values (*p* < 0.05). (**A**) before and (**B**) after incubation with oxLDLs; the upregulated genes are represented by orange dots and the downregulated genes are represented by blue dots.

RNA sequencing of M1-like macrophages was performed for four patients with FH (marked in Table 1, three patients with *LDLR* and one patient with *APOB* mutations) and four representatives of the control group (marked in Table 1). The complete lists of DEGs with nominal *p* < 0.05 from all comparisons are presented in Tables S2–S4. Analysis of the obtained data revealed 22 DEGs (six upregulated and 16 downregulated; Table S2) between the group of FH patients and the control group before oxLDL loading (basal condition), and significantly more after oxLDL loading—47 DEGs (39 upregulated and eight downregulated; Table S3) as visualized by volcano plot analysis (Figure 2).

The most upregulated gene in differentiated M1-like macrophages of patients with FH was *LYZ*, encoding lysozyme, an essential component of innate immunity. *LYZ* gene expression remained significantly elevated in FH M1-like macrophages after oxLDL exposure. The most downregulated gene in FH M1-like macrophages compared to controls was the *TMEM176B* encoding protein which plays a role in the regulation of NLRP3 inflammasome activation.

Incubation with oxLDLs resulted in more marked differences in macrophages between FH patients and control (Figure 2). Among the most upregulated genes in FH M1-like macrophages after oxLDL exposure were genes encoding markers of M1 polarization; particularly, *IDO1* and *GBP5* must be mentioned. According to Cytoscape pathway enrichment analysis, DEGs were mainly involved in biological pathways associated with the immune response (GO:0006955) and cellular stress response (GO:0006950) including transcription factor *STAT2* (important for IFNβ signaling) and *HPSE* encoding heparanase (participating in chemokine and cytokine release).

**Figure 3:**
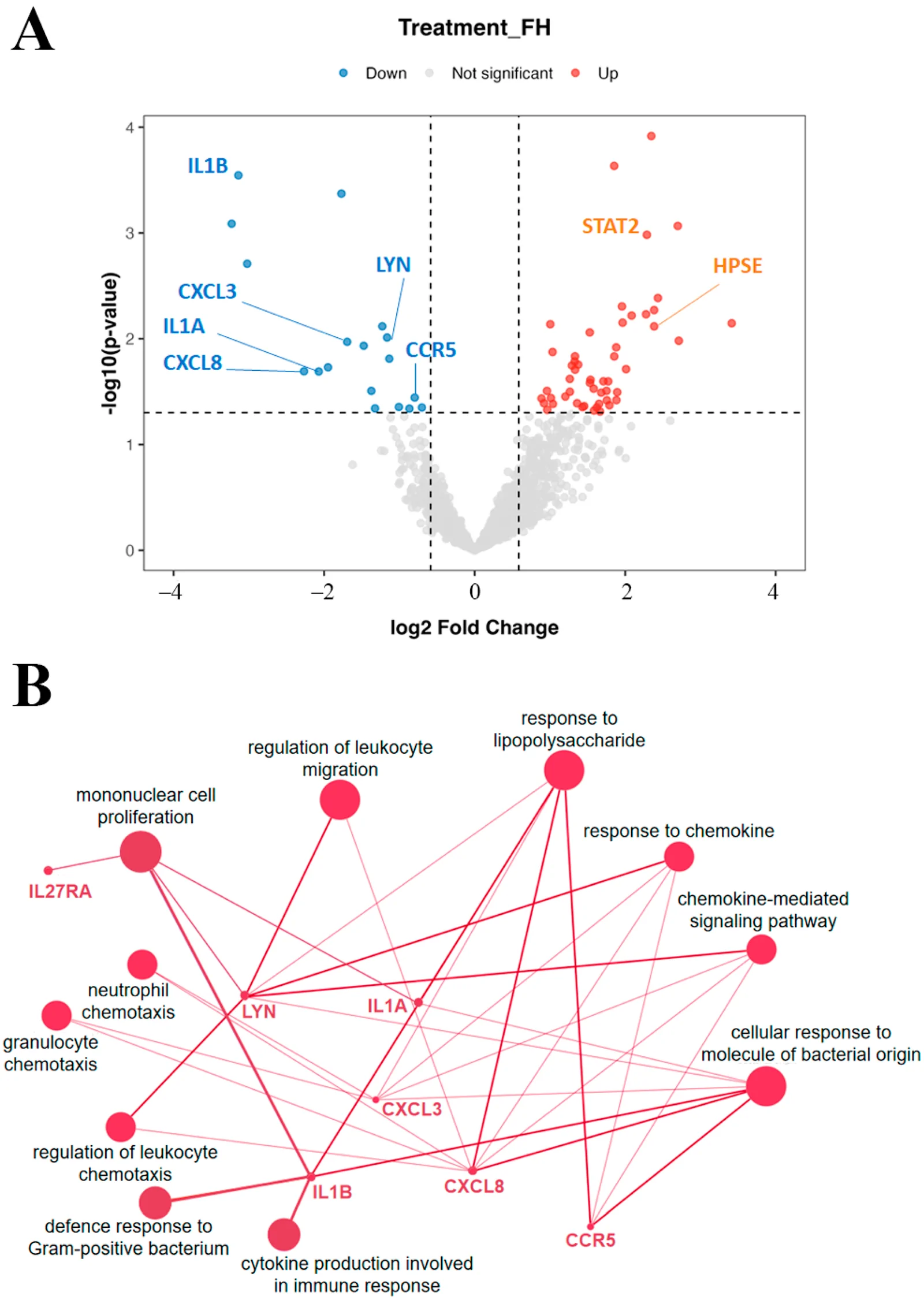
Effect of lipid accumulation in FH M1-like macrophages. (**A**) Volcano plot of mRNA expression in FH M1-like macrophages after oxLDL exposure versus baseline constructed using log2 fold-change (|FC| > 1.5) values and *p*-values (*p* < 0.05); the upregulated genes are represented by orange dots and the downregulated genes are represented by blue dots. (**B**) Functional enrichment analyses of inflammation-associated genes.

When analyzing the effect of macrophage transformation into foam cells during oxLDL exposure, it should be noted that, compared with the control group, FH M1-like macrophages were characterized by a greater number of genes changing their expression in response to lipid accumulation (65 DEGs, 47 upregulated and 18 downregulated, Table S4) (Figure 3A).

**Figure 4:**
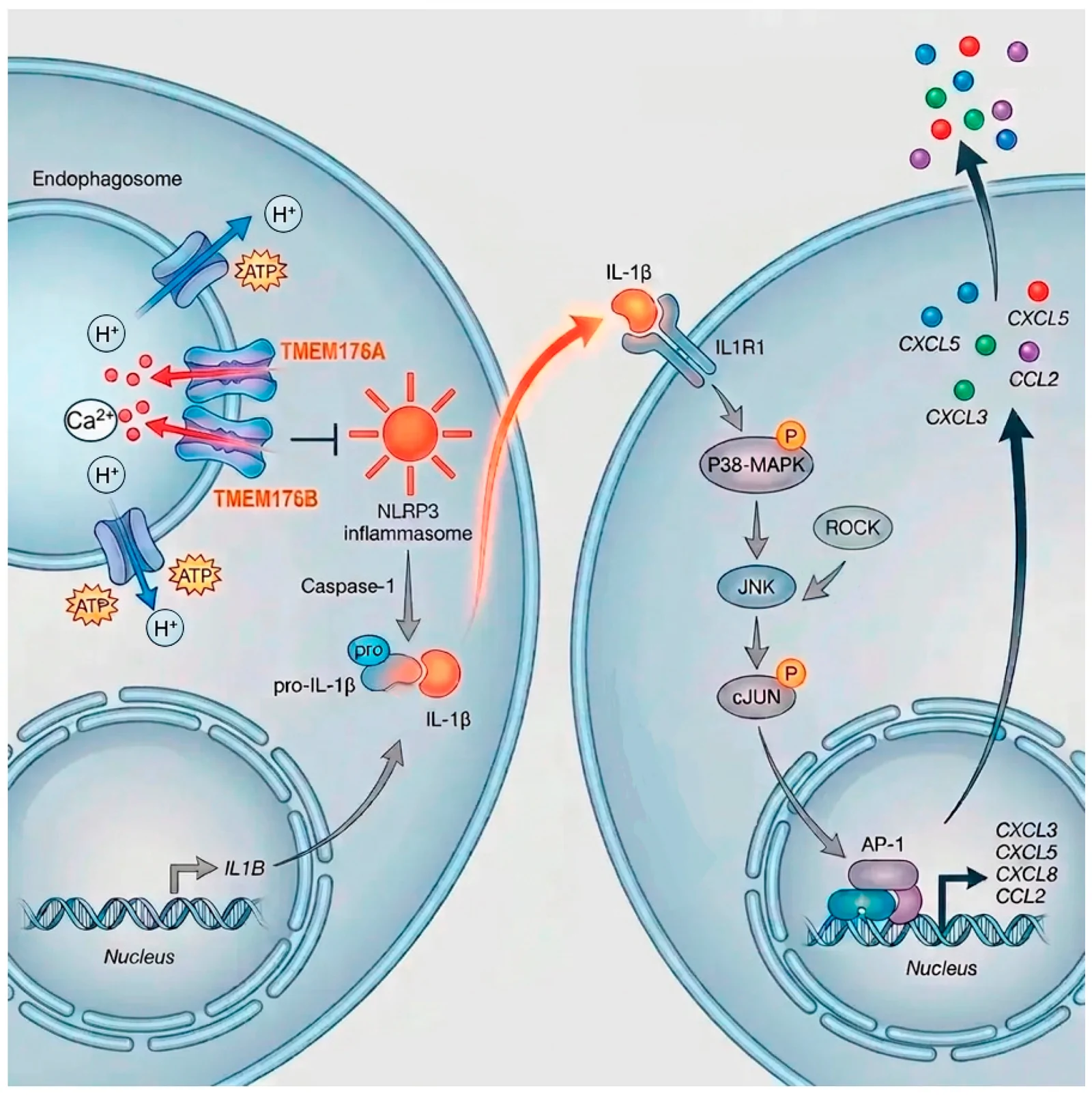
The interconnection between IL-1 and chemokine signaling pathways. TMEM176A and TMEM176B work as a cation channels, regulating cytoplasm calcium and subsequently calcium-dependent potassium influx required for NLRP3 inflammasome activation [21]. NLRP3 inflammasome, a cytosolic multiprotein complex, in activated state release caspase-1, which then processes pro-interleukin-1β to the active form that contributes to IL-1β secretion [23]. Further IL-1β regulates chemokine production via the MAPK signaling pathway [24,25]. This image was created with artificial intelligence.

Pathway enrichment analysis demonstrated that these genes are involved in IL-1 signaling pathways regulating immune responses, chemokine responses and production, immune cell migration, responses to lipopolysaccharide and cellular responses to molecules of bacterial origin, indicating potential dysregulation of these processes (Figure 3B). Accordingly, genes involved in these pathways were chosen for real-time PCR validation: *IL1A* and *IL1B*, *TMEM176A* and *TMEM176B* (involved in regulation of NLRP3 inflammasome and IL-1β maturation), main chemokines *CCL2* encoding monocyte chemoattractant protein-1 (MCP-1), *CXCL3*, *CXCL5*, *CXCL8* encoding IL-8, *CXCL10* (Figure 4), and additionally *LYZ*, *STAT2* and *HPSE* associated with the immune response.

### 3.2. IL-1 and Chemokine Signaling Pathway Activity Evaluation

Comparative analysis of the mRNA level of 12 genes mainly belonging to IL-1 and chemokine signaling pathways in M1-like macrophages was carried out in expanded groups (10 patients with FH and 10 healthy individuals (Table 1)) using real-time PCR. The gene list and primers are presented in Table S1. Additionally, IL-1β, MCP-1 and IL-8 concentrations were evaluated in culture mediums as well as in blood serum by ELISA.

**Figure 5:**
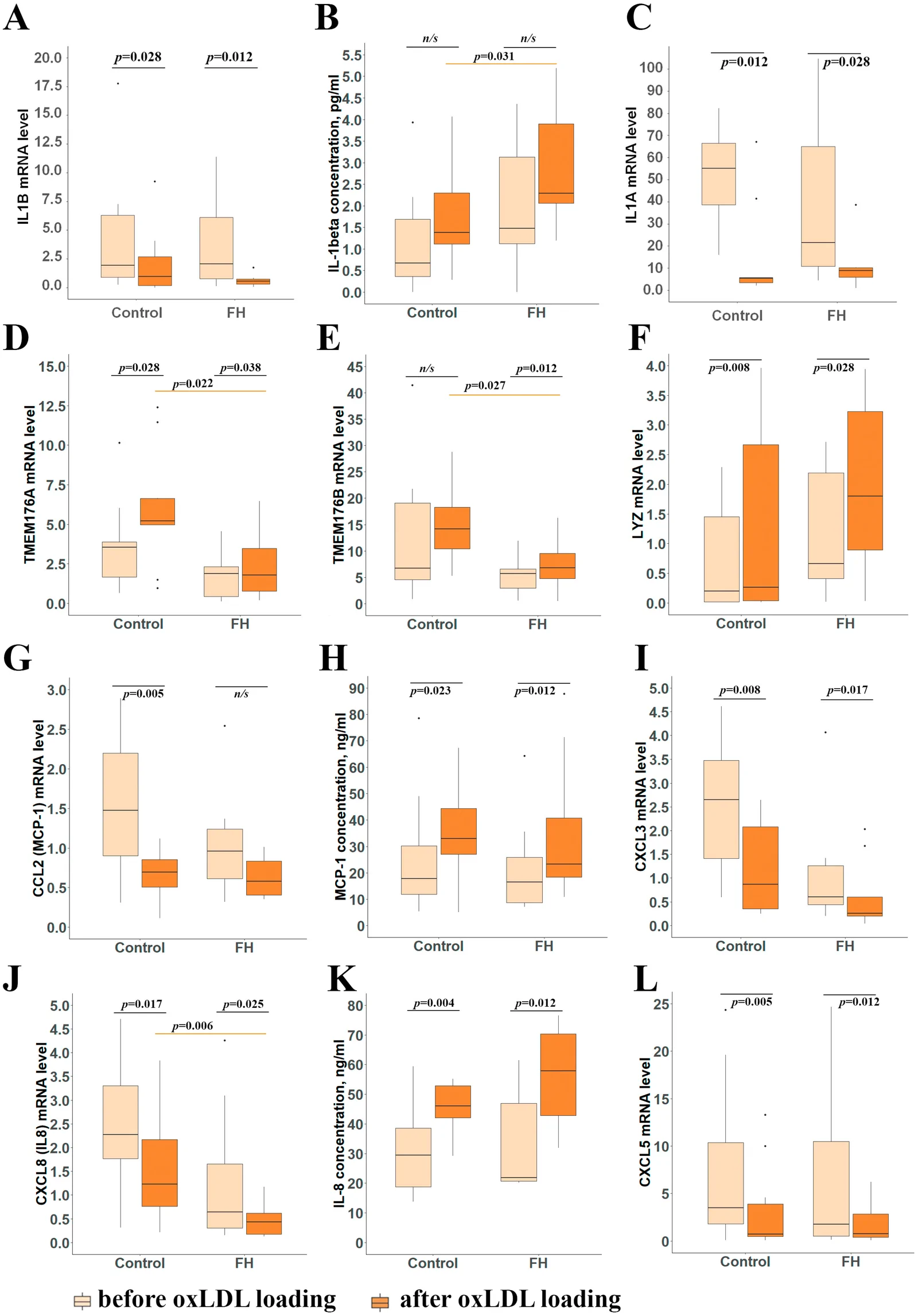
*IL1B*, *IL1A*, *TMEM176A*, *TMEM176B*, *LYZ*, *CCL2*, *CXCL3*, *CXCL5*, and *CXCL8* gene expression (relative mRNA levels and chemokine secretion levels) in M1-like macrophages in the studied groups. (**A**) *IL1B* mRNA level; (**B**) IL-1β secretion level; (**C**) *IL1A* mRNA level; (**D**) *TMEM176A* mRNA level; (**E**) *TMEM176B* mRNA level; (**F**) *LYZ* mRNA level; (**G**) *CCL2* mRNA level; (**H**) MCP-1 secretion level; (**I**) *CXCL3* mRNA level; (**J**) *CXCL8* mRNA levels; (**K**) IL-8 secretion level; (**L**) *CXCL5* mRNA level. Designations in the figure: n/s—non significant.

In accordance with transcriptomic data, *IL1A* and *IL1B* mRNA expression was reduced after oxLDL exposure both in FH patient’s and control M1-like macrophages (Figure 5A,C). At the same time, opposite to mRNA level, secretion of IL-1β tended to be elevated after incubation with oxLDL both in the FH group and in the control group. It is noticeable that some FH M1-like macrophages initially before oxLDL incubation had higher levels of IL-1β secretion. However, after oxLDL exposure, patient’s M1-like macrophages demonstrated increased IL-1β secretion levels compared to the control group indicating more a pronounced immune response (Figure 5B). Simultaneously, in accordance with transcriptomic data, FH M1-like macrophages were characterized by reduced *TMEM176B* as well its partner *TMEM176A* mRNA levels after incubation with oxLDLs compared to the control group (Figure 5D,E).

An extended set of chemokine gene expression changes was studied: *CXCL3* and *CXCL8* according to RNAseq data, and additionally *CCL2*, *CXCL5* and *CXCL10* to complete the full picture. *CXCL3*, *CXCL5*, and *CXCL8* mRNA levels were reduced upon oxLDL exposure both in the FH group and in the control group (Figure 5I,J,L). The *CCL2* mRNA level was significantly decreased only in control macrophages upon oxLDL exposure. Interestingly, *CXCL8* mRNA levels demonstrated a trend of being reduced (*p* = 0.068) in FH M1-like macrophages before oxLDL exposure, and were significantly reduced in FH macrophages compared to the control group after lipid loading (Figure 5J). *CXCL10* mRNA levels did not differ neither between groups nor depending on the addition of oxLDLs. At the same time, *CCL2* and *CXCL8* mRNA levels and corresponding chemokine production disagreed: MCP-1 and IL-8 secretion levels increased upon oxLDL loading both in FH patients and in the control group (Figure 5H,K). At the same time, neither IL-1β nor MCP-1 or IL-8 concentrations in blood serum differed between the FH group and the control group (Table 2).

The *LYZ* gene mRNA level was upregulated in M1-like macrophages upon oxLDL exposure in both groups (Figure 5F). Different expression of *STAT2* and *HPSE* genes was not confirmed.

## 4. Discussion

In the current study, we demonstrated that despite lipid-lowering therapy, the lipid-loaded M1-like macrophages of FH patients exhibited increased IL-1β secretion and reduced *TMEM176A* and *TMEM176B* mRNA levels compared to control macrophages. Here, we began by performing transcriptome analysis of M1-like macrophages of patients with genetically confirmed FH and healthy individuals which revealed that FH lipid loading results in more pronounced gene expression changes in M1-like macrophages with most DEGs relating to immune regulation. In lipid-laden M1-like macrophages of FH patients, the most upregulated genes were *IDO1* and *GBP5* which are markers of M1 polarization [19,26,27]. The most upregulated gene in FH M1-like macrophages before lipid loading was the *LYZ* gene, maintaining these changes after incubation with oxLDLs. The *LYZ* gene encodes lysozyme which acts as a natural defense mechanism by breaking down bacterial cell walls [28]. Further functional enrichment analysis of DEGs suggested dysregulation of multiple immune pathways linked to IL-1 signaling including the chemokine signaling pathway, immune cell chemotaxis and migration, mononuclear cell proliferation, responses to lipopolysaccharide and cellular responses to molecules of bacterial origin (Figure 3B). These findings are consistent with the hypothesis of “trained immunity” in FH.

IL-1 signaling acts as a core driver of vascular inflammation and primary initiator of atherosclerosis. IL-1 axis activation of M1-like macrophages in FH patients might predispose to accelerated development of atherosclerosis through an increased inflammatory response and may be linked to a preactivated state of blood monocytes in FH. Targeting inflammatory pathways, especially the NLRP3 inflammasome pathway and its regulated cytokine interleukin-1β, for the treatment of atherosclerotic diseases has been attracting attention for a long time [29]. Because of the established role of NLRP3 inflammasome in the development of atherosclerosis, therapeutic strategies aimed at inhibiting NLRP3 inflammasome activation (pharmacological inhibitors targeting NLRP3 inflammasome components including P2X7 receptor antagonists), as well as caspase-1 and anti-IL-1 antibodies, may have beneficial effects in protecting against inflammatory damage in atherosclerosis [29].

IL-1β is initially transcribed and translated into a pro-form that is biologically inactive [23]. The generation of mature IL-1β requires proteolytic removal of a pro-peptide sequence from the precursor. This process requires caspase-1 release from the activated NLRP3 inflammasome complex, after which mature IL-1β is secreted. Our study showed that *TMEM176A* and *TMEM176B* mRNA levels were reduced in lipid-loaded FH M1-like macrophages compared to that in the control group. This can explain the increased IL-1β secretion as it has been suggested that the TMEM176 complex inhibits the activation of the NLRP3 inflammasome, and as a result IL-1β maturation [23]. TMEM176A and TMEM176B are members of the MS4A (membrane-spanning 4-domain, subfamily A) family, and exhibit similar patterns of expression and are preferentially expressed in myeloid cells [30]. There are various data on the cellular localization of TMEM176 transporters: in cell mitochondria [31], in Golgi apparatus [32], on the endosomal membrane [23,33]. Thus, most of the available information concerns the intracellular localization of TMEM176s. TMEM176A and TMEM176B form multimers and work as a cation channel and, in response to adenosine triphosphate, inhibit the accumulation of calcium in the cytoplasm; notably, calcium-dependent potassium influx is required for inflammasome activation (Figure 4) [23]. A study of hepatic inflammation in mice showed that Tmem176b+ macrophages are predominantly anti-inflammatory, demonstrating a shift to the M2 phenotype [34]. *Tmem176b*−/−mice recruited significantly more neutrophils than wild-type animals [23]. Mouse *Tmem176b*−/−BMDCs as well as human *TMEM176B* knockout THP-1 cells demonstrated higher caspase-1 activation and secreted significantly higher levels of IL-1β [23,35]. Conversely, *TMEM176B* overexpression impaired IL-1β secretion by THP-1-differentiated macrophages [23]. These data are consistent with our results showing that control macrophages upon lipid loading demonstrated lower IL-1β secretion and higher *TMEM176A* and *TMEM176B* gene expression, making them more protected from atherosclerosis development. Recently, *TMEM176A* was identified as one of the hub genes showing a significantly lower expression in early-stage carotid atherosclerotic plaques compared to advanced-stage carotid atherosclerotic plaques which indicates that decreased activity of TMEM176s in early stages of atherogenesis could play a role in plaque initiation [36]. Interestingly, genome-wide association studies (GWASs) identified *TMEM176A* and *TMEM176B* as lipid-associated loci [37,38]. It was shown that *TMEM176B* deficiency promoted cholesterol accumulation in human glial cells derived from induced pluripotent stem cells suggesting TMEM176A and TMEM176B activity could be linked to dyslipidemia [39,40].

So, TMEM176A and TMEM176B play a significant role in the activation of inflammatory responses via the IL-1 signaling pathway, facilitating immune cell infiltration and differentiation [35,41]. It is known that IL-1 regulates chemokine production via the MAPK signaling pathway (Figure 4) [24,25]. In fact, several previous transcriptomic studies of FH peripheral blood mononuclear cells and their progenitors underlined that chemotaxis and cytokine and chemokine signaling are among the most upregulated pathways [8,42,43]. At the same time, Ye et al. revealed that genes encoding members of the MAPK family (such as *MAPK1*, *MAP3K1*, and *MAPK9*) were differentially expressed in FH blood cells [44]. Plasma IL-1 concentration was shown to be increased in hypercholesterolemia, as well as circulating levels of MCP-1 (encoded by the *CCL2* gene) and IL-8 (encoded by the *CXCL8* gene) which are positively correlated with TC, LDL-C and LDL’s structure protein apoprotein B plasma concentrations in FH [45,46,47]. MCP-1 and IL-8 produced largely by monocytes and macrophages are likely to be central chemokines playing a role in atherosclerosis development in FH. MCP-1 is highly expressed in human atherosclerotic lesions and is considered to be a key player in monocyte recruitment to the arterial wall [48]. IL-8 promotes leukocyte adhesion and migration to the endothelium, exacerbating inflammation and plaque formation. Thus, hyperlipidemia promotes the expansion of circulating myeloid cells, including monocytes and neutrophils, that may lead to elevated plasma concentrations of the chemokines MCP-1 and IL-8 and upregulated expression of the corresponding receptors, CCR2 and CXCR2 [8,49]. As it was shown earlier for THP-1 macrophages, oxidized lipids cause increased IL-8 secretion [50]. Thus, increased MCP-1 and IL-8 secretion by M1-like macrophages observed in our study should have been expected. But opposingly, in our study, gene expression of *CXCL8* as well as *CXCL3* and *CXCL5* was reduced in M1-like macrophages upon oxLDL loading and we can assume that CXCL3 and CXCL5 secretion levels may also be inappropriate.

Still, there is no clear picture of these chemokine’s gene expression across different macrophage subtypes (M1/M2) in FH. In our study, mRNA levels of *CXCL3*, *CXCL5* and *CXCL8* were reduced in M1-like macrophages after incubation with oxLDLs in both FH and control groups. At the same time, secretion of MCP-1 and IL-8 increased, in contrast to the observed decrease in their mRNA levels. This discrepancy may be attributable to an early peak of their induction, which was missed at a selected time (24 h). Such regulation could be particularly important as these chemokines bind to the same receptor CXCR2.

For example, Martin-Fuentes et al. reported that increased expression of the *CXCL3* gene was observed only in macrophages of FH patients with tendon xanthomas during the early stage of incubation with oxLDLs, whereas *CXCL8* mRNA levels tended to increase only after prolonged incubation (18 h) [51]. However, this study lacked a control group and used a heterogeneous macrophage population. Consistently with our findings, Lappalainen et al. demonstrated that oxLDL loading leads to a decrease in *CCL2* and *CXCL8* gene expression in macrophages obtained by monocyte differentiation in the presence of M-CSF [52]. The authors proposed that the transformation of macrophages into foam cells may reduce their proinflammatory and atherogenic potential. However, this conclusion remains controversial, as the study did not assess the level of cytokine and chemokine secretion. In contrast, Willemsen et al. showed that exposure to acetylated LDL resulted in upregulation of *IL1B* and *CXCL8* mRNA levels in non-polarized M-CSF human macrophages [53].

Data regarding chemokine CXCL5 are also contradictory. Elevated blood CXCL5 concentrations were associated with a higher risk of hypercholesterolemia [54]. On the other hand, in coronary artery disease, plasma CXCL5 levels were shown to be negatively correlated with disease severity [55]. This may be due to its effect on angiogenesis and the formation of a more ramified microvascular bed: in patients with chronic coronary artery disease, higher concentrations of the angiogenic chemokine CXCL5 in the blood correlate with a greater quantity of coronary collaterals [56]. The results of the study by Rousselle et al. indicate that CXCL5 may play a protective role in atherogenesis: CXCL5 inhibition increased the accumulation of foam cells in plaque, while exogenous administration of CXCL5 limited foam cell formation by increasing the expression of the ABCA1 transporter and cholesterol efflux from M2 macrophages [57]. In fact, recent data indicate chemokines may play a role not only in new immune cell attraction but also in the regulation of macrophage subpopulations in the atherosclerotic plaque microenvironment. For example, Yu et al. showed that the chemokine CXCL3 plays an important role in the positive regulation of M2b macrophage polarization within plaque, which may contribute to the suppression of inflammation and the formation of stable plaques [58]. Similar data were obtained for CXCL8, but in the tumor microenvironment [59].

Overall, our data is in agreement with previous reports showing the proinflammatory and promigratory phenotype of monocytes and their potential to differentiate into M1-like macrophages in FH [2,3,8,11,12]. Monocytes from patients with FH demonstrate enhanced cytokine production and capability to migrate into arterial intima and appropriate proinflammatory transcriptional reprogramming [2,4,9]. Our data showed that lipid-laden M1-like macrophages differentiated from monocytes of treated FH patients are predisposed to an accelerated immune response via interleukin-1β and this may be related to reduced *TMEM176A* and *TMEM176B* gene expression and leading NLRP3 inflammasome activation. Therefore, taking into account the specific pattern of *TMEM176A* and *TMEM176B* gene expression in myeloid cells, they may be considered as potential therapeutic targets in dyslipidemia and atherosclerosis. Further research is needed to clarify the role of TMEM176A and TMEM176B in the pathogenesis of FH associated inflammation.

The study has several limitations. The sample sizes were relatively small and the in vitro experiments conducted do not fully reflect the same M1 macrophage population in the atherosclerotic plaque microenvironment due to the complex local macrophage heterogeneity. On the other hand, studies of specific macrophage subsets are relevant and informative for identification of disease-driving mechanisms and search for targeted therapies.

## 5. Conclusions

Our data suggest that monocytes of FH patients are initially more inflammatory-oriented, and therefore lipid-laden M1-like macrophages differentiated from monocytes of FH patients are predisposed to an accelerated immune response via interleukin-1β, likely due to reduced *TMEM176A* and *TMEM176B* gene expression. Additionally, analysis of our data in conjunction with literature data suggests that regulation of chemokine gene expression and secretion may be very complex in the context of the plaque microenvironment and various macrophage subtypes.

## Figures and Tables

**Table 1:** Characteristics of the participants.

**N**	**Person ID, Sex (M/F), Age**	Genetic Variant *	**Rosuvastatin** **/** **Ezetemib Dose, mg**	**TC (mmol/L)**	**LDL-C (mmol/L)**
**FH Patients**					
1	LM139, M, 32	*LDLR* rs121908027 chr19:11105557TGG [1] NM_000527.5:c.651TGG [1] p.(Gly219del)	20/10	8.1	6.0
2	FH26, M, 47	*LDLR* rs121908038 chr19:11113293T>A NM_000527.5:c.1202T>A p.(Leu401His)	20/10	9.2	5.6
3	LM109, F, 31	*LDLR* rs137929307 chr19:11116928G>A NM_000527.5:c.1775G>A p.(Gly592Glu)	10/10	8.1	4.6
4	1017, F, 27	*APOB* rs5742904 chr2:21006288C>T NM_000384.3:c.10580G>A p.(Arg3527Gln)	20/10	8.5	5.2
5	1041, F, 22	*LDLR* rs121908029 chr19:11105588G>T NM_000527.5:c.682G>T p.(Glu228Ter)	20/-	10.6	8.5
6	LM38 M, 42	*LDLR* rs879254671 chr19:11106638C>A NM_000527.5:c.768C>A p.(Asp256Glu)	20/10	7.6	4.9
7	LM116, F, 40	*LDLR* rs761954844 chr19:11110697G>A NM_000527.5:c.986G>A p.(Cys329Tyr)	20/-	10.2	7.9
8	1014, M, 54	*LDLR* rs765696008 chr19:11113268G>A NM_000527.5:c.1187-10G>A	40/10	14.9	11.1
9	LM146 F, 30	*LDLR* rs875989938 NC_000019.10:g.11120412G>A NM_000527.5:c.2030G>A p.(Cys677Tyr)	10/10	9.1	7.4
10	FM15, F, 48	*APOB* rs5742904 chr2:21006288C>T NM_000384.3:c.10580G>A p.(Arg3527Gln)	10/10	8.3	6.4
**Control group**					
**N**	**Person ID, sex (M/F), age**	Genetic Variant *	**Rosuvastatin** **/** **Ezetemib dose, mg**	**TC (mmol/L)**	**LDL-C (mmol/L)**
1	N, M, 34	-	-	3.4	2.2
2	B, M, 42	-	-	5.2	3.1
3	M, F, 41	-	-	5.2	3.2
4	S, F, 40	-	-	3.1	1.7
5	K, F, 50	-	-	5.0	2.5
6	U, F, 39	-	-	5.1	2.7
7	I, M, 27	-	-	4.7	3.1
8	O, F, 45	-	-	5.2	3.2
9	T, M, 31	-	-	3.2	1.7
10	G, F, 39	-	-	3.8	1.7

* Gene name, rsID, GRCh38 genomic coordinate, variant position, amino acid substitution.

**Table 2:** Demographic and biochemical characteristics of the studied groups.

Parameter	FH Patients	Control Group	*p*
Age, years	36 (22–54)	39 (27–50)	>0.05
Sex (M/F)	4/6	4/6	>0.05
Serum TC, mmol/L	8.8 (7.6–14.9)	4.8 (3.1–5.2)	0.000
Serum LDL-C, mmol/L	6.2 (4.6–11.1)	2.5 (1.7–3.2)	0.000
Serum IL-1β, pg/mL	4.6 (0–11.8)	3.2 (0–24.2)	>0.05
Serum MCP-1, ng/mL	181.2 ± 83.6	157.7 ± 76.0	>0.05
Serum IL-8, ng/mL	8.3 (0–89.6)	5.8 (0–18.5)	>0.05

Data are presented as median (minimum–maximum) in the case of non-normal data distribution or as mean ± SD in the case of normal distribution.

## Data Availability

The original contributions presented in this study are included in the article. Further inquiries can be directed to the corresponding authors.

## Supplementary Materials

The following supporting information can be downloaded at: https://www.mdpi.com/article/10.3390/cimb48080769/s1 [60,61].

## Abbreviations

The following abbreviations are used in this manuscript:

BM	Bone marrow
CVD	Cardiovascular disease
DEG	Differentially expressed gene
DLCN	Dutch Lipid Clinic Network
FBS	Fetal bovine serum
FH	Familial hypercholesterolemia
HDL-C	High-density lipoprotein cholesterol
HSPCs	Hematopoietic stem and progenitor cells
IL-1β	Interleukin 1β
IL-8	Interleukin 8
INFβ	Interferon β
INFγ	Interferon γ
LDL	Low-density lipoprotein
LDL-C	Low-density lipoprotein cholesterol
LPS	Lipopolysaccharide
MCP-1	Monocyte chemoattractant protein-1
M-CSF	Macrophage colony-stimulating factor
NLRP3	NOD-, LRR- and pyrin domain-containing protein 3
oxLDLs	Oxidized low-density lipoproteins
TC	Total cholesterol
