# Supplementary material for: Lipid-Laden M1-like Macrophages of Familial Hypercholesterolemia Patients Are Characterized by Increased Interleukin-1β Secretion and Reduced TMEM176A and TMEM176B Gene Expression
Source: Curr Issues Mol Biol. 2026 Jul 29;48(8):769. doi: 10.3390/cimb48080769 (PMC13511675; doi:10.3390/cimb48080769)
Supplement: Supplementary file 1 [file cimb-48-00769-s001.zip › cimb-4429340-supplementary.pdf]

Macrophage phenotype was confirmed by flow cytometry and real-time PCR according to Jaguin et al [19].

Flow cytometry

M0 and M1 macrophages were stained with monoclonal mouse anti-human CD14 (Biolegend, USA) and CD80 (Elabscience, China) antibodies. Cells were analyzed using a BD FACSCanto II flow cytometer (BD Biosciences, USA) (Figure S1). M1-like macrophages cells are characterized by an increased proportion of CD80+ positive cells consistently with previous reports [19,60].

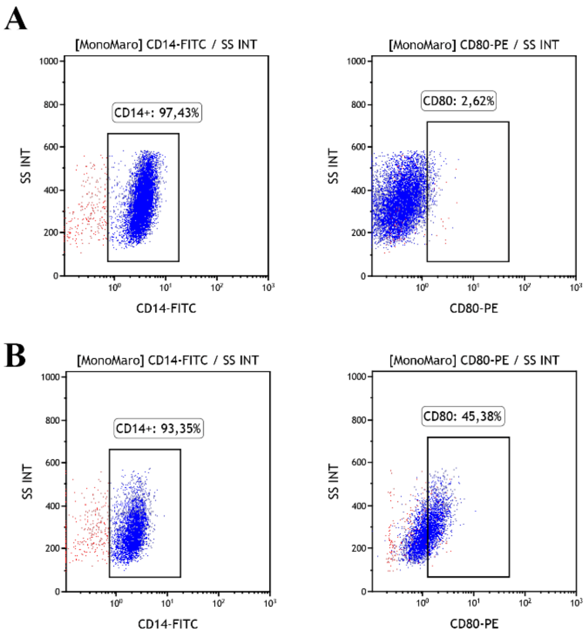

Figure S1. Flow cytometry analysis of CD14 and CD80 surface expression before (A) and after (B) LPS/IFN-γ stimulation of M-CSF human monocyte-derived M0-macrophages.

mRNA expression of markers in human unpolarized and polarized monocyte-derived macrophages

Due to the limited amount of biomaterial (especially in the case of FH patients) we assessed a set of mRNA expression markers of M1 and M2 polarization by real-time PCR. 6 markers of macrophage polarization were selected: CD68 – as common macrophage marker, IDO1, CXCL10 – as M1 polarization markers, PPARγ, TGF-β, HMOX1 – for M2 polarization markers. These M1 and M2 markers were chosen as the most distinguishing M0, M1 and M2 subtypes according [19]. The primers and probes sequences used in this work are presented in the Table S1.

M1-like macrophages were characterized by significantly increased expression of M1 genes (CXCL10 and IDO1) and decreased expression of M2 genes (PPARG, TGFB1 and HMOX1) genes compared to M0 macrophages (Figure S2). A moderate increase in the CD68 gene expression level was also observed in M1-like macrophages compared to M0 macrophages (Figure S2) consistent with the data that LPS/IFN-γ stimulation usually increase the fraction of cells expressing CD68 on their surface [61].

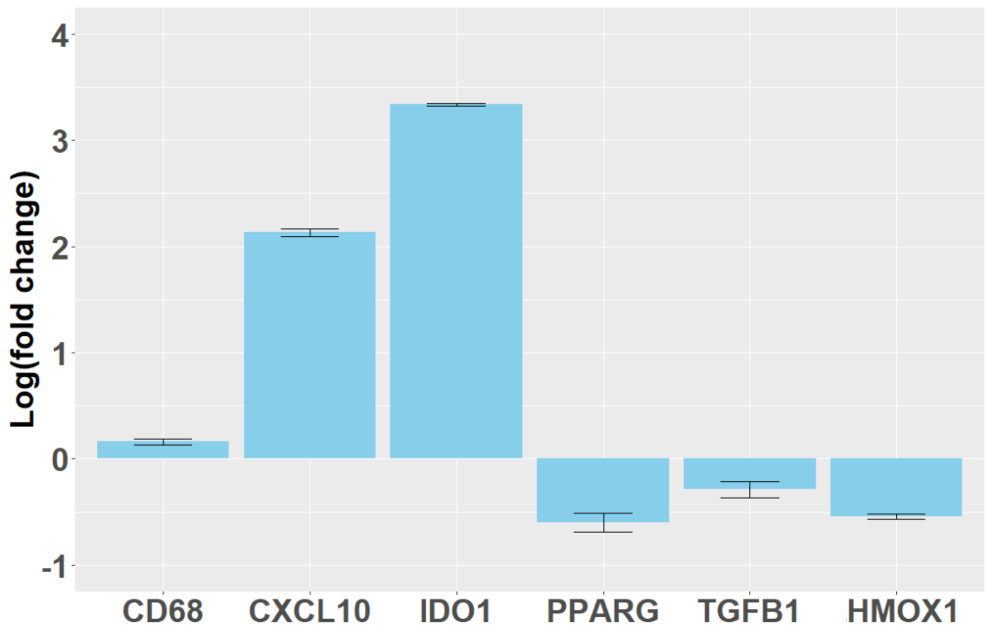

Figure S2. mRNA expression of putative M1 and M2 polarization markers in M1-like macrophages. M0-macrophages were activated with LPS/IFN-γ to obtain M1 polarization. Data are expressed relatively to corresponding mRNA levels found in M0 M0-macrophages, arbitrarily set at the value of 1, and are the means ± SEM of three independent experiments. $p<0.05$ for all genes.

Table S1. Real-time PCR primers and probes sequences

Gene	5'-3' Sequence of forward primer, reverse primer and Taq-man probe*
Macrophage polarization confirmation step	
CD68	CATGGCGGTGGAGTACAATG<br>GATCTCGAAGGGATGCATTC<br>CGCAGCACAGTGGACATTCTCGGC
CXCL10	CTCCAGTCTCAGCACCATGA<br>GCAGGTACAGCGTACAGTTC
IDO1	GCCTTGCACGTCTAGTTCTG<br>GAGAGTTGGCAGTAAGGAACAG
PPARG	GATGTCTCATAATGCCATCACGTT<br>GGATTCAGCTGGTCGATATCACT<br>**FAM**-CCAACAGCTTCTCCTTCTCGGCCTG-**BHQ1**
TGFB1	TTTGATGTCACCGGAGTTGT<br>GTGTTATCCCTGCTGTCACA
HMOX1	CGACAGCATGCCCCAGGATT<br>TGAAGCCGTCTCGGGTCAC
RNAseq validation step	
CCL2	AGCAAGTGTCCCAAAGAAGC<br>TGGAATCCTGAACCCACTTC
CXCL3	TCAAGAACATCCAAAGTGTGAA<br>ATGCGGGGTTGAGACAAG<br>**FAM**-CCCAAACCGAAGTCATAGCCA-**BHQ1**
CXCL5	GATCCAGAAGCCCCTTTTCT<br>GAAACTTTTCCATGCGTGCT<br>**FAM**-TGGACGGTGGAAACAAGGAAA-**BHQ1**
CXCL8	CCTTTCCACCCCAAATTTATC

	CCTTGGGGTCCAGACAGAG<br>**FAM**-TGCGCCAACACAGAAATTATTGTAAA-**BHQ1**
IL1A	TCATTTCATTGGCGTTTGAG<br>AGACAGATGATCAATGGAGGAA<br>**FAM**-CCTGAAGAACTGTTACAGTGAAAATGA-**BHQ1**
IL1B	ACAGATGAAGTGCTCCTTCCA<br>CCATGGCCACAACAACTGAC
TMEM176A	TACACCCTCCTCGTCACCTC<br>ATTACCACCCCGTTTCTCGT<br>**FAM**-GACAGGGGCTGTGGCTGTG-**BHQ1**
TMEM176B	GAGAACCAATGGCAGAAGGA<br>GACACAGACAGCCAGGAACA<br>**FAM**-AGCTTACATGCAGATGCTGAGGAAGT-**BHQ1**
LYZ	CAAAACCCCAGGAGCAGTTA<br>TTGCACAAGCTACAGCATCA<br>**FAM**-GCAGTGCTTTGCTGCAAGATAACAT-**BHQ1**
HPSE	CGTGCAAGGTTCAAAGAGAA<br>AAGGATAGGGTAACCGCAAG<br>**FAM**-CACAAACACTGACAATCCAAGGT-**BHQ1**
STAT2	GGAACGGAGGAAATACCTGA<br>CAGCCCTAGTTCCAGCTCTA<br>**FAM**-CTAATAGACAGGTGGATGAACTGCAA-**BHQ1**
Reference genes	
ACTB	CGTGCTGCTGACCGAGG<br>ACAGCCTGGATAGCAACGTACA<br>**HEX**-CCAACCGCGAGAAGATGACCCAGAT-**BHQ1**
RPLP0	GATCAGGGACATGTTGCTGG<br>GACTTCACATGGGGCAATGG<br>**ROX**-CAATAAGGTGCCAGCTGCTGC-**BHQ2**

*for SYBR Green only primer pairs sequences are provided

Table S2. M1-like macrophages before incubation with oxLDLs (baseline): complete list of DEGs from comparison "FH patients vs control group"

	Gene	log2FoldChange	Module	pvalue
1	***LYZ***	**2,876658809**	**2,876658809**	**0,003598944**
2	*TCTA*	-1,509984739	1,509984739	0,00581443
3	*SMAP2*	0,975656126	0,975656126	0,016104939
4	***TMEM176B***	**-2,357530569**	**2,357530569**	**0,019596526**
5	*FLVCR2*	-0,911645604	0,911645604	0,020605259
6	*ARF3*	0,982098406	0,982098406	0,022973776
7	*ITGA5*	-1,254010678	1,254010678	0,022998257
8	*JAK3*	-1,365869593	1,365869593	0,023850823
9	*MSN*	1,077249647	1,077249647	0,02626338
10	*ARF5*	-1,389450771	1,389450771	0,027754214

11	*GABPB2*	-1,144050143	1,144050143	0,029409191
12	*ACADVL*	-1,316607789	1,316607789	0,031416185
13	*ILRUN*	-1,275624684	1,275624684	0,033988672
14	*FOXRED1*	-1,230852275	1,230852275	0,03408431
15	*CTSC*	-1,618921019	1,618921019	0,035075928
16	*RPL37*	2,250937809	2,250937809	0,042782499
17	*ETFB*	-1,243589285	1,243589285	0,04427348
18	*DGKA*	-1,439229774	1,439229774	0,045416578
19	*TINF2*	-1,072834975	1,072834975	0,046528317
20	*CD3D*	-1,346567923	1,346567923	0,047146532
21	*CAPNS1*	-2,019432037	2,019432037	0,048475349
22	*LAPTM5*	1,251937806	1,251937806	0,049399756

Table S3. M1-like macrophages after incubation with oxLDLs: complete list of DEGs from comparison "FH patients vs control group"

	Gene	log2FoldChange	Module	pvalue
1	*ARF3*	1,303456651	1,303456651	0,001410348
2	*MR1*	1,174925707	1,174925707	0,001472269
3	*CD2BP2*	2,511814119	2,511814119	0,001885601
4	***IDO1***	**2,893946044**	**2,893946044**	**0,002297866**
5	***STAT2***	**2,089674646**	**2,089674646**	**0,002729016**
6	*RPL36AP37*	1,905166309	1,905166309	0,003534349
7	*NUB1*	1,165794106	1,165794106	0,004452095
8	*DFFA*	-1,203360778	1,203360778	0,005320915
9	*SERPING1*	2,227983198	2,227983198	0,005730233
10	*YARS1*	-0,982236701	0,982236701	0,007123646
11	*YPEL3*	1,977667967	1,977667967	0,008333354
12	*TNFRSF14*	2,257257178	2,257257178	0,008787512
13	*EEF2*	1,864648548	1,864648548	0,01075317

14	*IL27RA*	1,900012156	1,900012156	0,012171115
15	*GBP2*	1,589084735	1,589084735	0,014478345
16	*VEZF1*	1,294332928	1,294332928	0,014843271
17	*NSFL1C*	-0,710670289	0,710670289	0,016622548
18	*SSRP1*	-1,018669535	1,018669535	0,017262392
19	*SZRD1*	1,447115718	1,447115718	0,018419855
20	*SLC44A2*	1,982747837	1,982747837	0,019375977
21	*VPS4B*	1,603395707	1,603395707	0,021154083
22	*PSMA1*	1,538084747	1,538084747	0,024454314
23	*MT1G*	2,502193375	2,502193375	0,02517363
24	*ATOSB*	1,810092291	1,810092291	0,025198651
25	***GBP5***	**2,604465337**	**2,604465337**	**0,026082491**
26	*KLHL12*	-1,041098706	1,041098706	0,026270023
27	*LAPTM5*	1,336480575	1,336480575	0,026797417
28	*FLOT1*	-1,212486358	1,212486358	0,027001492
29	*SRSF2*	0,992413289	0,992413289	0,027109658
30	***LYZ***	**2,060447672**	**2,060447672**	**0,027873**
31	*RPS12*	2,239262888	2,239262888	0,028002559
32	*LAP3*	1,299688214	1,299688214	0,02928174
33	*APOL2*	1,101447599	1,101447599	0,030759302
34	*RNF149*	1,039372916	1,039372916	0,031150048
35	*EIF3B*	-1,114330833	1,114330833	0,033457665
36	*HLA-DRB1*	-2,18471492	2,18471492	0,034258454
37	***HPSE***	**1,879168168**	**1,879168168**	**0,03521548**
38	*WDR13*	1,665149846	1,665149846	0,035553233
39	*PPA1*	0,987389949	0,987389949	0,037089186
40	*FHIP2B*	1,954026207	1,954026207	0,037545832
41	*FPR1*	1,615519268	1,615519268	0,04112442

42	*ENSA*	1,118437262	1,118437262	0,041794813
43	*ATXN10*	1,092233978	1,092233978	0,042548222
44	*IFIT3*	1,991521489	1,991521489	0,0457222
45	*DNMT1*	1,178350164	1,178350164	0,04576311
46	*RASSF5*	0,844586087	0,844586087	0,049183328
47	*WARS1*	1,34639995	1,34639995	0,049373934

Table S4. FH M1-like macrophages: complete list of unique DEGs from comparison "after incubation with oxLDLs vs baseline".

	Gene	log2FoldChange	Module	pvalue
1	*CTSD*	2,346857356	2,346857356	0,000121032
2	*HMOX1*	1,853589516	1,853589516	0,000231969
3	***IL1B***	**-3,139036516**	**3,139036516**	**0,000284986**
4	*DHCR24*	-1,770181868	1,770181868	0,000424553
5	*TNFAIP6*	-3,228023873	3,228023873	0,000817393
6	*CD2BP2*	2,698359693	2,698359693	0,000857364
7	***STAT2***	**2,288178887**	**2,288178887**	**0,001040093**
8	*THBS1*	-3,022165918	3,022165918	0,001952862
9	*PUF60*	2,43479127	2,43479127	0,004117752
10	*VPS4B*	1,956899273	1,956899273	0,004945294
11	*RPL13*	2,384357002	2,384357002	0,005355197
12	*TYK2*	2,276681579	2,276681579	0,005870421
13	*IL27RA*	2,085463545	2,085463545	0,006037782
14	*FERMT3*	1,96543576	1,96543576	0,007042645
15	*PSME1*	3,414906143	3,414906143	0,007135333
16	*FLVCR2*	1,004874677	1,004874677	0,007287182
17	*HMGCS1*	-1,226950571	1,226950571	0,007623462
18	***HPSE***	**2,384488885**	**2,384488885**	**0,007633057**
19	*CTNS*	1,529321557	1,529321557	0,008715208

20	*LYN*	-1,163463722	1,163463722	0,009732828
21	*C1QA*	2,712117907	2,712117907	0,010438561
22	***CXCL3***	**-1,693947517**	**1,693947517**	**0,010689485**
23	*NDRG2*	-1,473968292	1,473968292	0,011652397
24	*YPEL3*	1,880669832	1,880669832	0,012060354
25	*RSRC1*	1,034712823	1,034712823	0,013335934
26	*NELFE*	1,332455023	1,332455023	0,0146575
27	*OSCAR*	1,854472488	1,854472488	0,014676278
28	*KLHL12*	-1,13385076	1,13385076	0,015440426
29	*FOXRED1*	1,328714577	1,328714577	0,016377685
30	*KIAA0930*	1,375100973	1,375100973	0,017569217
31	*CPNE1*	1,291990945	1,291990945	0,017907568
32	*SLC39A8*	-1,94817125	1,94817125	0,01862465
33	*TNFRSF14*	2,011398536	2,011398536	0,019361189
34	*ILRUN*	1,333545244	1,333545244	0,019607484
35	***CXCL8***	**-2,267242889**	**2,267242889**	**0,020301227**
36	***IL1A***	**-2,071404598**	**2,071404598**	**0,020405959**
37	*CNPPD1*	1,264102323	1,264102323	0,023955797
38	*TCN2*	1,539233329	1,539233329	0,024439489
39	*VAT1*	1,710949412	1,710949412	0,025246144
40	*WDR13*	1,774192069	1,774192069	0,025275292
41	*GRN*	1,531873334	1,531873334	0,02609799
42	*NCF4*	1,581324233	1,581324233	0,029647979
43	*HLA-E*	1,751646646	1,751646646	0,031093309
44	*ERAP2*	-1,372417825	1,372417825	0,031108035
45	*MGAT1*	0,960631659	0,960631659	0,031140152
46	*ETFB*	1,264738076	1,264738076	0,031716633
47	*PSMB9*	1,894103485	1,894103485	0,032004688

48	*ATF4*	1,682248763	1,682248763	0,032323885
49	*WDR1*	1,204734698	1,204734698	0,035203864
50	*CCR5*	-0,796712868	0,796712868	0,036006345
51	*MLEC*	1,013507273	1,013507273	0,036312501
52	*RETREG2*	0,888286129	0,888286129	0,036645646
53	*POLRMT*	1,882880809	1,882880809	0,038061761
54	*SLC44A2*	1,75607839	1,75607839	0,038180804
55	*PRMT2*	0,920450343	0,920450343	0,040674006
56	*AUP1*	1,360737414	1,360737414	0,040733095
57	*UPF3B*	1,652523586	1,652523586	0,04124513
58	*APOL2*	1,040183186	1,040183186	0,041309206
59	*TRPV2*	1,454656009	1,454656009	0,043352897
60	*IDI1*	-1,006639011	1,006639011	0,044192305
61	*DBNL*	1,43222984	1,43222984	0,044329298
62	*ATOSB*	1,622515643	1,622515643	0,044644028
63	*TOX4*	-0,700755445	0,700755445	0,044651445
64	*RNF144B*	-1,323415626	1,323415626	0,0455418
65	*DFFA*	-0,867212704	0,867212704	0,045752521

Supplementary references

60. Bertani, F.R.; Mozetic, P.; Fioramonti, M.; Iuliani, M.; Ribelli, G.; Pantano, F.; Santini, D.; Tonini, G.; Trombetta, M.; Businaro, L.; et al. Classification of M1/M2-polarized human macrophages by label-free hyperspectral reflectance confocal microscopy and multivariate analysis. *Sci. Rep.* **2017**, *7*, 8965. https://doi.org/10.1038/s41598-017-08121-8

61. Tedesco, S.; Bolego, C.; Toniolo, A.; Nassi, A.; Fadini, G. P.; Locati, M.; Cignarella, A. Phenotypic activation and pharmacological outcomes of spontaneously differentiated human monocyte-derived macrophages. *Immunobiology*. **2015**, *220*, 545–554. https://doi.org/10.1016/j.imbio.2014.12.008
